# Monitoring Specific IgM and IgG Production Among Severe COVID-19 Patients Using Qualitative and Quantitative Immunodiagnostic Assays: A Retrospective Cohort Study

**DOI:** 10.3389/fimmu.2021.705441

**Published:** 2021-09-03

**Authors:** Jamil A. Al-Mughales, Tareq J. Al-Mughales, Omar I. Saadah

**Affiliations:** ^1^Department of Clinical Laboratory Medicine, Diagnostic Immunology Division, King Abdulaziz University Hospital, Jeddah, Saudi Arabia; ^2^Department of Medical Microbiology and Parasitology, Faculty of Medicine, King Abdulaziz University, Jeddah, Saudi Arabia; ^3^Faculty of Medicine, King Abdulaziz University, Jeddah, Saudi Arabia; ^4^Department of Pediatrics, Faculty of Medicine, King Abdulaziz University, Jeddah, Saudi Arabia

**Keywords:** severe, COVID-19, SARS-CoV-2, IgG, IgM, immunoglobulins, Saudi Arabia

## Abstract

The purpose of this study is to monitor specific anti-severe acute respiratory syndrome coronavirus 2 (anti-SARS-CoV-2) IgG and IgM antibody production in patients with severe forms of coronavirus disease 2019 (COVID-19) using various commercially available quantitative and qualitative tests. The sera of 23 confirmed COVID-19 patients were processed for anti-SARS-CoV-2 IgG and IgM detection. Three different immunoassays, viz. Abbott Architect^®^ SARS-CoV-2 IgG assay, and two quantitative tests, ANSH^®^ SARS-CoV-2 and AESKULISA^®^ SARS-CoV-2 Nucleocapsid Protein (NP), were performed and the results pooled, from diagnosis to serum collection. Seroconversion rates were computed for all 3 assays, and possible correlations were tested using the Pearson correlation coefficient and Cohen’s kappa coefficient. Overall, 70 combinations of qualitative and quantitative IgG and IgM results were pooled and analyzed. In the early phase (0-4 days after diagnosis), in all tests, IgG seroconversion rates were 43%-61%, and increased in all tests gradually to 100% after 15 days. The Pearson correlation coefficient showed a strong positive relationship between the qualitative IgG test results and both quantitative IgG tests. IgM detection was inconsistent, with maximal concentrations and seroconversion rates between 10-15 days after diagnosis and slight-to-fair agreement between the two quantitative immunoassays. There was no significant association between mortality with IgG or IgM seroconversion or concentrations. Patients with severe COVID-19 develop an early, robust anti-SARS-CoV-2 specific humoral immune response involving IgG immunoglobulins. Further comparative studies are warranted to analyze the value of serological testing in predicting the severity of COVID-19 and detecting prior exposure.

## 1 Introduction

Following several months of an unprecedented global struggle against the COVID-19 pandemic, caused by severe acute respiratory syndrome coronavirus 2 (SARS-CoV-2), the long-term efficacy of the deployed preventive, diagnostic, and therapeutic strategies remain uncertain. The feasibility and effectiveness of radical measures, such as public lockdowns and mandatory social distancing, must be reconciled with social and economic costs. Several studies have highlighted significant variance in adherence to the use of barrier measures, such as face masks, especially in vulnerable settings, such as healthcare institutions (1, 2). Other reports underscore the negative socioeconomic impact of lockdowns and the difficulty in implementing social-distancing rules and enforcing control measures, particularly in underprivileged populations (3, 4). In light of the above concerns, it is suggested that at this stage management of the pandemic may require reappraisal. Forecasting effective detection and quarantining of individuals with active disease, coupled with enhancement of the long-term immunity of a population, should be prioritized.

Promising management strategies may include systematic screening using sensitive and specific methods to encompass all cases and prevent further dissemination of the virus in the general population. The current diagnostic strategy relies on the detection of viral RNA in upper and lower respiratory airways, using molecular methods, notably reverse transcription-polymerase chain reaction (rt-PCR) tests designed against the virus envelope and RNA-dependent polymerase regions (5).

The rt-PCR method is theoretically recognized as highly sensitive and specific. However, it is technically demanding and costly, and has a relatively long turnover time. Other limitations include common risk for false-negative results owing to frequent incorrect sampling in practice, which can lead to repeated testing, and result in additional expenses (6, 7). Furthermore, with rt-PCR, viral RNA can be detected only during the active phase of infection, which is estimated between the second day after symptom onset and 10-15 days following a first positive result (8, 9). Combined, these limitations of the test can lead to reduced sensitivity, which, in the context of the present pandemic, can result in a substantial number of false-negative results, thus, undermining the overall management strategy and jeopardizing the security of the healthcare system and population safety (10). Thus, it is becoming more commonly recommended to integrate the detection of specific SARS-CoV-2 antibodies within management strategy towards improving the screening, diagnosis, and follow-up of patients—both at the healthcare institutional level and in the general population (11). Accordingly, it is clinically relevant to determine previous infection or exposure to the virus and to understand the course of specific antibody production. However, it is still undetermined whether specific antibody production grants protective immunity and prevents eventual reinfection, or, by contrast, whether antibodies act as virus entry enhancers, previously observed in related coronaviruses (12, 13). If the latter hypothesis is correct, increased antibody production may be related to severe COVID-19 and so would not be of prognostic value.

At the time when the present study was conducted, many of these scientific questions were pending. A few IgG and IgM immunoassay kits have obtained marketing authorizations, notably in Saudi Arabia, yet they were not validated clinically in the local patients. Broader research criteria were necessary to determine the clinical and epidemiological utility of SARS-CoV-2 serodiagnosis, including the synthesis of data from various institutions and population categories. Among these questions was the kinetics of IgM and IgG production in patients with severe form of COVID-19.

This study examined specific anti-SARS-CoV-2 IgG and IgM antibody production in a sample of hospitalized patients with severe COVID-19, using three different immunoassays including one IgG qualitative test and two IgG and IgM quantitative tests. The researchers analyzed the progression over time of the immunoglobulins levels and compared the results of the tests by looking for possible correlations between the respective quantitative results and potential agreement with qualitative interpretations.

## 2 Methods

### 2.1 Design and Setting

This was a retrospective cohort study conducted at King Abdulaziz University Hospital, Jeddah, Saudi Arabia, between 25 April and 7 July, 2020. All procedures were carried out in agreement with the ethical principles for medical research involving human subjects set out by the World Medical Association. The study protocol was approved by the Research Ethics Committee of King Abdulaziz University (Reference No. 478-20).

### 2.2 Population

This study included adults admitted as inpatients for management and treatment of severe COVID-19, which was defined in our institution as any case having a documented pneumonia with decreased oxygen saturation, with or without signs of respiratory distress. Participants were diagnosed based on evocative clinical presentation and epidemiological context, and confirmed with at least one positive rt-PCR (Abbott Real-Time SARS-COV-2 assay; USA) test carried out between 25 April and 25 May, 2020. Immunocompromised patients, patients with mild disease, and patients that were treated as outpatients were excluded.

### 2.3 Immunoassays

#### 2.3.1 Procedure and Methods

Sera of the COVID-19 patients were collected and stored at -20°C. Sera of eligible patients were prepared at room temperature for processing. The reagents were prepared as per the respective manufacturer protocols for the 5 immunoassays, including: Abbott Architect SARS-CoV-2 IgG assay (SARS-CoV-2 nucleocapsid protein, Abbott, Illinois, USA); SARS-CoV-2 IgG ELISA AL-1001-r and SARS-CoV-2 IgM ELISA (μ-Capture) AL-1002-r assays (SARS CoV-2 nucleocapsid protein and spike protein, ANSH Labs, Texas, USA); and AESKULISA^®^ SARS-CoV-2 Nucleocapsid Protein (NP) IgG and AESKULISA^®^ SARS-CoV-2 NP IgM assays (AESKU DIAGNOSTICS, Wendelsheim, Germany). Abbott SARS-CoV-2 IgG assay consists of a chemiluminescent microparticle immunoassay (CMIA) used for qualitative detection in human serum of specific Sar-CoV-2 IgG antibodies, directed against the nucleoprotein of the virus. Serum samples were processed on the Abbott Architect *i*2000SR instrument (Abbott, Illinois, USA) using the FDA-approved SARS-CoV-2 IgG kit assay according to the manufacturer insert.

ANSH Labs SARS-CoV-2 IgG/IgM assays are enzyme-linked immunosorbent assays (ELISA) tests used for quantitative and semi-quantitative detections in human serum. The assays detect SARS-CoV-2-specific IgG and IgM antibodies against two capture protein antigens, including spike (S) and nucleocapsid (NP). The patient serum sample was diluted (1:101), distributing 10 μL of serum into 1 mL of the IgG or IgM sample diluent in the culture tube, as appropriate, then harmonized in a centrifuge. The sample was kept aside for 10 minutes. Following this, 100 μL of the calibrators and diluted serum samples were pipetted to the appropriate wells and incubated for 45-60 minutes at 37°C, without shaking. Each well was then aspirated and washed 5 times with the wash solution. 100 μL of prepared SARS-CoV-2 IgG or IgM Antigen-Enzyme Conjugate Solution, as appropriate, was added to each well and incubated for 45-60 minutes at 37°C, without shaking. Each well was aspirated and washed 5 times with the wash solution. Subsequent to this, 100 μL of tetramethylbenzidine (TMB) chromogen solution was added to each well, using a precision pipette, and the wells were incubated at room temperature for 8-12 minutes; the incubation time was optimized by visual monitoring of the color. The stopping solution was then added (100 μL) and the absorbance of the solution in the wells was read within 5 minutes using a microplate reader set to 450 nm.

AESKULISA^®^ SARS-CoV-2 NP IgG and IgM assays are qualitative and quantitative ELISA tests used for the detection of specific SARS-CoV-2 IgG and IgM antibodies in human serum. These tests use recombinant NP only from SARS-CoV-2 as the recombinant antigens. The antibody testing was processed according to the ELISA principles and according to the AESKULISA kit manufacturer insert.

#### 2.3.2 Results Interpretation

For Abbott SARS-CoV-2 IgG assay, index values and the corresponding qualitative results were collected and analyzed as shown by the instrument; that is, a positive result was indicated by a value >1. For ANSH Labs SARS-CoV-2 IgG and IgM assays, the results were calculated by plotting the data on a log vs. log scale using a linear regression curve fit; the results were considered positive for a sample concentration >12 Arbitrary Unit (AU)/mL and negative if < 10 AU/mL. For AESKULISA^®^ SARS-CoV-2 NP IgG and IgM assays, reading was carried out using a semiautomated method, assisted by the AESKU READER & Software. The output data is a result value ranging between 3 and 100 U/mL, with a cut-off value of 8-12 U/mL, above which the result was considered positive.

### 2.4 Data Collection

In addition to immunoassay results, data and the dates of the respective specimen collection, demographic, and clinical data of the patients were collected from the hospital electronic system. These included information related to age; gender; medical history; presenting symptoms including systemic (fever, headache, etc.), respiratory (cough, shortness of breath, chest pain), and extra-respiratory (nausea, vomiting, diarrhea, etc.) symptoms; and final patient outcome (survival *vs.* mortality).

### 2.5 Statistical Methods

Statistical analysis was performed with the Statistical Package for the Social Sciences version 21.0 for Windows (SPSS Inc., Chicago, IL, USA). Frequencies and percentages were used for categorical variables, and means and standard deviations (SDs) or medians were used for continuous variables. Wilcoxon signed-ranked test was incorporated to analyze the paired change in IgG index, using Abbott qualitative assay between the first and the second testing of each patient. Mann-Whitney U test was used to compare IgG titer and IgG and IgM levels between recovered and deceased patients at all time points and using all methods separately; results are presented as median values with 75^th^ centile (P75), as well as the corresponding level of statistical significance. Repeated-Measure ANOVA (RM-ANOVA) was used to analyze the change in ABBOTT IgG titers between the first and second testing, as a function of the patient outcome (recovery versus death); the effect size of time, outcome and time*outcome were estimated by calculation of Eta squared.

All patient qualitative and quantitative immunoassay results were pooled with respect to the time of the result, calculated as the number of days from the date of diagnosis, indicated by the first positive rt-PCR to the date of specimen collection. The Pearson correlation coefficient calculated bivariate correlations between the 3 assays using their respective IgG and IgM concentrations and indices as expressed through raw values. Seroconversion rates were calculated as the percentage of positive results in all 3 assays; and in quantitative assays, i.e. ANSH Labs SARS-CoV-2 IgG/IgM AESKULISA^®^ SARS-CoV-2 NP IgG/IgM assays, the equivocal results were taken as negative. The degree of agreement between various assays was analyzed using Cohen’s kappa coefficient to test corresponding serological results (positive or negative) of each pair of tests. The kappa value (k) was interpreted as no agreement (< 0), slight (0-0.20), fair (0.21-0.40), moderate (0.41-0.60), substantial (0.61-0.80) and perfect (0.81-1.0) agreement (14). Furthermore, considering the time of the result (with reference to the date of diagnosis), the pooled assay results were categorized into 4 periods: 1) early (0-4 days after the diagnosis); 2) second (5-10 days); third (10-15 days); and late (> 15 days). The progression through time of mean antibody concentration and index values were compared over the 4 periods using a one-way ANOVA, and the respective positivity rates were calculated and depicted using bar charts. A *P* value of <0.05 was set as statistically significant.

## 3 Results

### 3.1 Participant Characteristics

The study included 23 rt-PCR-confirmed COVID-19 patients, with moderately severe to severe forms of disease presentation, requiring hospitalization. The mean age (± SD) was 53.74 (± 12.20) years, and 18 out of the 23 patients (78%) were male. Of the 23 patients, 12 had comorbidities, including diabetes (8), hypertension (7), and renal diseases (4). The majority of the 23 patients presented with respiratory (15) and systemic (15) symptoms, while 6 had extra-respiratory symptoms. Fourteen of the 23 patients ultimately deceased (**Table 1**).

**Table 1:** Demographic and clinical data of the participants.

Parameter	Category	Frequency	Percentage
Age (years)	Mean, SD	53.74 ± 12.2	
Gender	Male	18	78.3
	Female	5	21.7
Medical history	Diabetes mellitus	8	34.8
	Hypertension	7	30.4
	Renal disease	4	17.4
	*Dialysis*	*2*	*8.7*
	*Chronic kidney disease*	*1*	*4.3*
	*Hydronephrosis*	*1*	*4.3*
	Other		
	*Cancer*	*1*	*4.3*
	*Heart failure*	*1*	*4.3*
Presenting Symptoms	***Systemic^a^***	***17***	***73.9***
	Fever	17	73.9
	Anorexia	1	4.3
	Fatigue	1	4.3
	Headache	1	4.3
	Night sweating	1	4.3
	***Respiratory^a^***	***17***	***73.9***
	Cough	14	60.9
	Chest pain	3	13.0
	Shortness of breath	3	13.0
	***Extra-respiratory^a^***	***6***	***26.1***
	Nausea/Vomiting	4	17.4
	Diarrhea	3	13.0
Final outcome	Mortality	14	60.9
	Survival	9	39.1

a A patient may have more than one symptom in the same category.

### 3.2 Specific IgG Qualitative Testing

**Figure 1:**
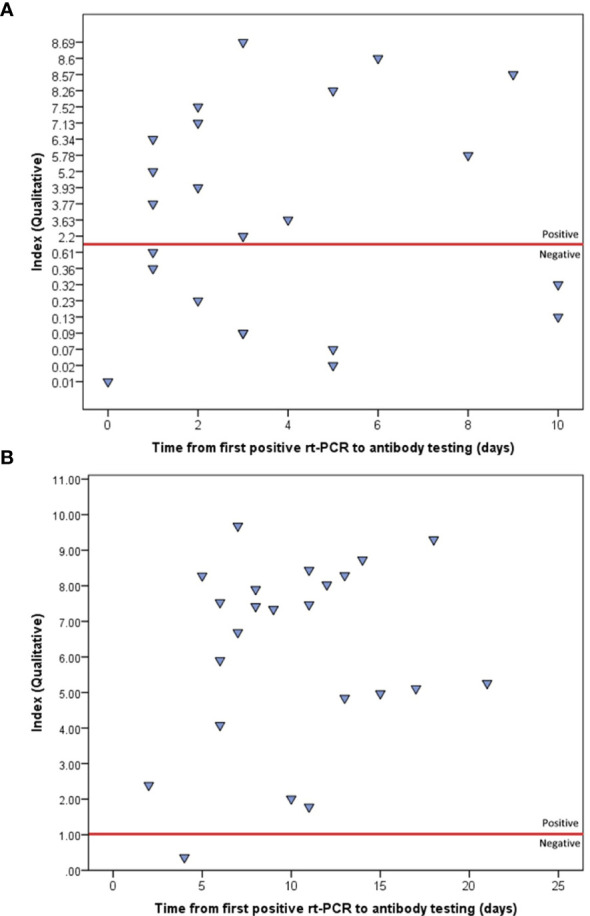
Indices of the first **(A)** and second **(B)** qualitative IgG testing, using ABBOTT method, as plotted by the time from first positive rt-PCR. The red line represents the positivity threshold (index>1).

**Figure 2:**
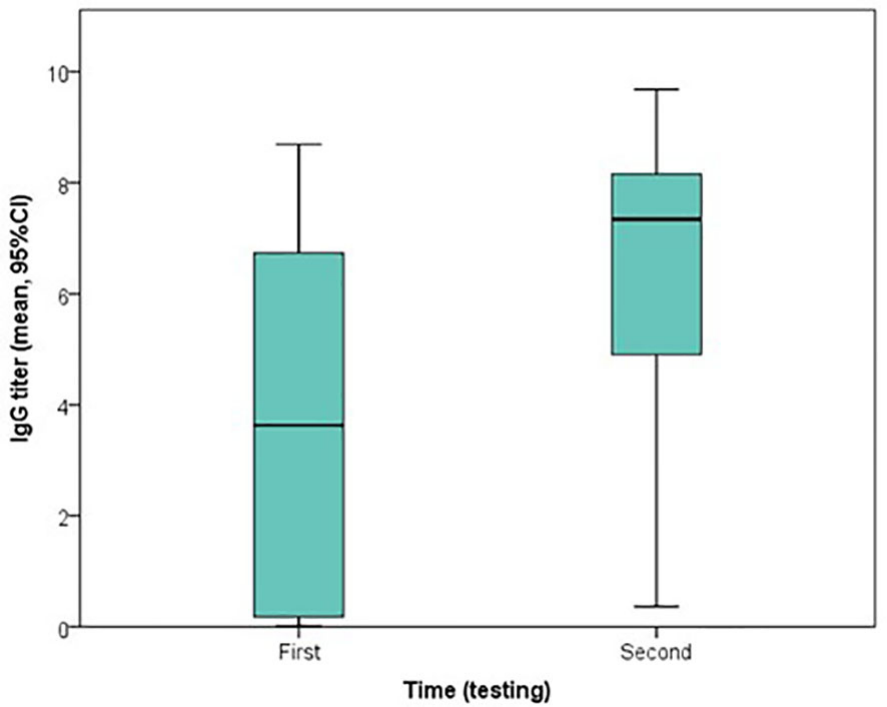
Boxplot of the change in indices of qualitative IgG testing, using ABBOTT method, from the first to second testing.

All 23 patients had at least two qualitative IgG test results, at two different time points. Depending on the case, the first testing was carried out from 0 to 10 days (median=3 days) after the first positive rt-PCR, which returned positive for 13 patients (56.5%). The second testing was carried out from 2 to 21 days (median=10 days) after the first positive rt-PCR, which returned positive for 22 (95.7%) patients. Furthermore, an overall increase in IgG indices was observed from the first (median index=3.63) to second testing (median index=7.34), and the difference was statistically significant (Wilcoxon signed ranked test, *P*<0.001) (**Figures 1**, **2**).

### 3.3 Specific IgG and IgM Quantitative Testing

Seventeen of the 23 patients (73.9%) had at least one quantitative IgG and IgM testing using the two methods simultaneously. The results of the first quantitative IgM and IgG testing for these 17 patients are depicted with respective time points in **Supplemental Table 1**. Of these 17 patients, 14 had at least 3 repeated measurements at different time points, while the remainder had fewer measurements. However, qualitative IgG testing was carried out 3 times for 19 of the 23 patients and 2 times for the remaining 4 patients, using the two tests simultaneously.

### 3.4 Association of Antibody Titers and Patient Outcome

**Figure 3:**
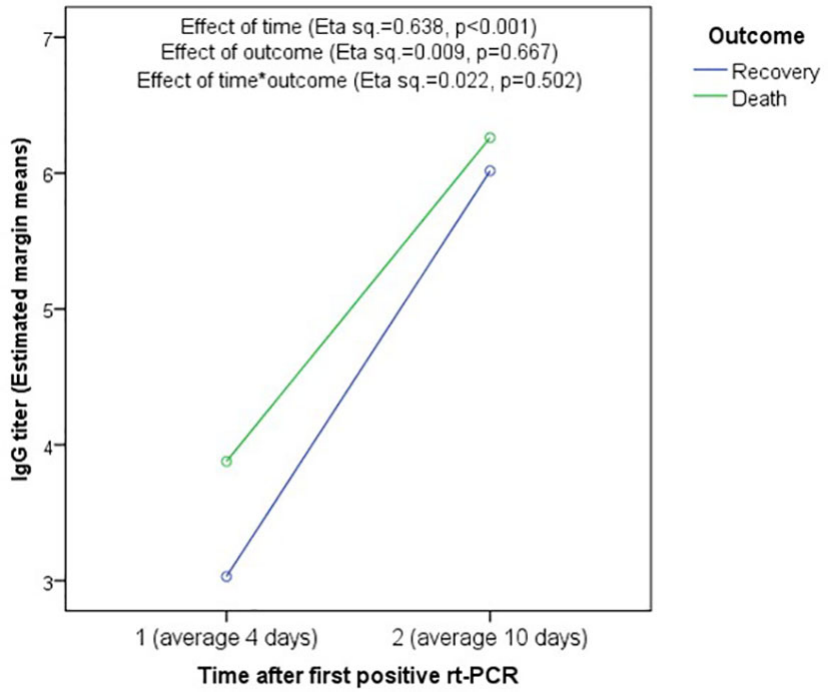
Association between mortality and change in IgG titer using Repeated-measure ANOVA. There is no significant association of IgG titer with outcome (Eta squared = 0.009, p = 0.667) or time*outcome (Eta squared = 0.022, p = 0.502). However, the effect of time was significant showing significant increase in IgG titers between the first and second testing in both recovered and deceased patients.

No statistically significant difference in IgG or IgM antibody levels was observed between deceased and survived patients, at any time point using both raw data (**Supplemental Table 2**) and pooled data (**Supplemental Figure 1**), and RM-ANOVA showed no significant effect of outcome (Eta squared=0.009, p=0.667) or time*outcome (Eta squared = 0.022, p=0.502) in the change in IgG titers between the first and second testing (**Figure 3**). Further, no statistically significant difference was found between recovered and deceased patients regarding seroconversion rates at the first (55.6% versus 57.1%, p=1.000) and second testing (88.9% versus 100.0%, p=0.391), respectively (**Supplemental Figure 2**).

### 3.5 Correlations Between the Different Testing Methods

Overall, 70 combinations of qualitative and quantitative IgG and IgM testing results at various timepoints were pooled and analyzed. The Pearson correlation coefficient was used to analyze the raw values of IgG and IgM levels of various assays, shown in **Table 2**; the agreement between the respective serological statuses (positive versus negative/equivocal) are depicted in **Table 3**. The results show a strong positive correlation between qualitative IgG index and IgG level from the first method (AESKU) (Pearson correlation coefficient [R]=0.87, *P*<0.001) and between levels of both quantitative IgM methods (R=0.79, *P*<0.001). The two quantitative IgG methods were weakly correlated (R=0.42, *P*=0.002). By considering the serological status (positive vs. negative or equivocal), the qualitative IgG method showed substantial agreement with both quantitative IgG methods (k = 0.74 and 0.66) while the serological statuses determined by the two quantitative methods were in perfect agreement (k=0.86). A fair agreement was found between the two quantitative IgM methods (k=0.25).

**Table 2:** Pearson correlation testing between qualitative IgG indices and quantitative IgG and IgM titers using different methods (pooled data).

Ig Method		IgG qualitative (ABBOTT)	IgG quantitative 1 (AESKU)	IgM quantitative 1 (AESKU)	IgG quantitative 2 (ANSH)	IgM quantitative 2 (ANSH)
IgG qualitative (ABBOTT)	R	–	0.87	0.54	0.35	0.60
	*P* value		<.001*	<.001*	0.019*	<0.001*
IgG quantitative 1 (AESKU)	R	0.87	–	0.41	0.42	0.38
	*P* value	<0.001*		0.003*	0.002*	0.006*
IgM quantitative 1 (AESKU)	R	0.54	0.41	–	-0.07	0.79
	*P* value	<0.001*	0.003*		0.623	<0.001*
IgG quantitative 2 (ANSH)	R	0.35	0.42	-0.07	–	0.09
	*P* value	0.019*	0.002*	0.623		0.544
IgM quantitative 2 (ANSH)	R	0.60	0.38	0.79	0.09	–
	*P* value	<0.001*	0.006*	<0.001*	0.544	

R, Pearson’s correlation coefficient; * statistically significant result (P < 0.05).

Bold values mean statistically significant P values.

**Table 3:** Agreement between different IgG and IgM testing methods (pooled data).

Ig Method		IgG Qualitative (ABBOTT)	IgG quantitative 1 (AESKU)	IgM quantitative 1 (AESKU)	IgG quantitative 2 (ANSH)	IgM quantitative 2 (ANSH)
IgG qualitative (ABBOTT)	Kappa	–	0.74	0.18	0.66	0.42
	*P* value		<0.001*	0.033*	<0.001*	<0.001*
IgG quantitative 1 (AESKU)	Kappa	0.74	–	0.30	0.86	0.53
	*P* value	<0.001*		0.003*	<0.001*	<0.001*
IgM quantitative 1 (AESKU)	Kappa	0.18	0.30	–	0.23	0.25
	*P* value	0.033*	0.003*		0.010*	0.049*
IgG quantitative 2 (ANSH)	Kappa	0.66	0.86	0.23	–	0.50
	*P* value	<0.001*	<0.001*	0.010*		<0.001*
IgM quantitative 2 (ANSH)	Kappa	0.42	0.53	0.25	0.50	–
	*P* value	<0.001*	<0.001*	0.049*	<0.001*	

*Statistically significant result (p < 0.05).

Bold values mean statistically significant P values.

### 3.6 Progression Over Time of Seroconversion Rates

**Figure 4:**
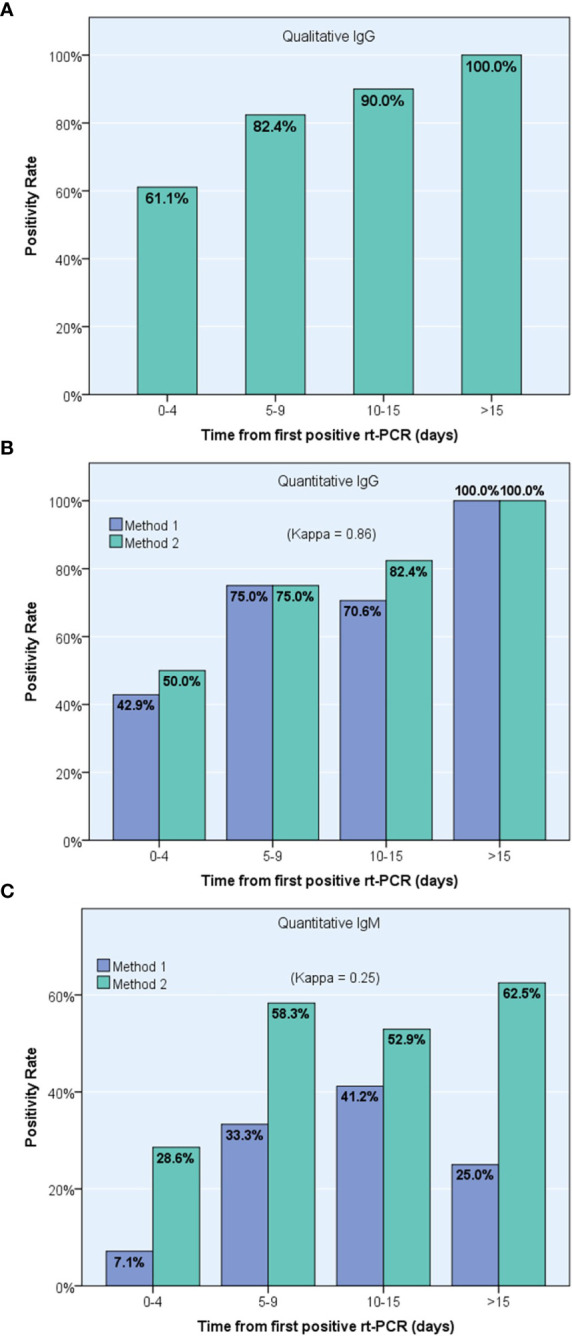
Progression over time of positive detection of specific anti-Sars-Cov-2 antibodies using pooled results from 23 confirmed COVID-19 patients, including one IgG qualitative immunoassay **(A)** and two different IgG **(B)** and IgM **(C)** quantitative immunoassays, ANSH (Method 1) and AESKU (Method 2).

By dividing the time from diagnosis to serum collection into 4 periods, the researchers observed a gradual increase in IgG detection rates using both the qualitative method (from 61.1% positive detection cases in the early period [0-4 days] to 100% in the last period [>15 days], **Figure 4A**) and the two quantitative methods (from 42.9% and 50.0% in the first period to 100% in both methods in the last period, respectively, **Figure 4B**). However, quantitative IgM positivity rate fluctuated over time, reaching a maximum of 41.2% and 62.5% for ANSH and AESKU, respectively (**Figure 4C**).

### 3.7 Progression Over Time of IgM and IgG Levels

A gradual increase in the levels of IgG production was observed from early to late phases of the disease, both using the qualitative method (*P*<0.001) and the two quantitative methods (*P*=0.021 for AESKU and *P*=0.002 for ANSH, respectively). However, IgM peaked in the third period, 10-15 days after first rt-PCR, and then declined in both quantitative methods (**Table 4**).

**Table 4:** Progression over time of IgM and IgG levels by testing method (pooled data).

Time from first positive rt-PCR (days)	IgG Qualitative (ABBOTT)		Quantitative 1 (AESKU)				Quantitative 2 (ANSH)			
	Mean	SD	IgG		IgM		IgG		IgM	
			Mean	SD	Mean	SD	Mean	SD	Mean	SD
0-4	3.09	2.89	41.72	46.98	3.43	4.09	239.57	728.26	8.28	5.56
5-9	6.10	3.04	59.49	44.42	14.65	13.90	329.60	769.18	17.16	12.25
10-15	6.02	2.95	71.71	44.25	27.46	34.86	422.09	881.74	26.45	29.42
>15	7.01	1.58	100.00	0.00	9.19	12.13	1786.55	1336.14	14.96	9.10
*P* value	0.001*		0.021*		0.029*		0.002*		0.073	

Test used, OneWay ANOVA; *statistically significant difference (P <0.05).

Bold values mean statistically significant P values.

## 4 Discussion

Since the outbreak of the COVID-19 pandemic, the immunogenic features of SARS-CoV-2 has elicited keen interest among the scientific community. This area of research constitutes the key to understanding whether the infection provides permanent immunity and whether an eventual vaccine would be efficient in preventing the infection. SARS-CoV-2 is an encapsulated, single-stranded RNA virus, belonging to the genus *Betacoronavirus*, subgenus *Sarbecovirus*, from the family of *Coronaviridae*. Sharing approximately 80% similarity with SARS-CoV-1 and 96% similarity with bat coronavirus, the genome of SARS-CoV-2 encodes 4 structural proteins, including the spike (S), envelope (E), membrane (M), and nucleocapsid (NP), besides other non-structural proteins. Early studies suggested that the S protein of SARS-CoV-2, with its two subunits S1 and S2, was the principal target of neutralizing immunoglobulins, and consequently the best target for vaccines (15–17). As an effect of a higher degree of sequence similarity in S2 compared with S1 subunits, a cross-reactive cellular immunogenicity was suggested between the S2 subunit of SARS-CoV-1 and SARS-CoV-2. Similarly, cross-reactivity in humoral immune response was identified between the two viruses, including monoclonal and polyclonal neutralizing antibodies directed against S2 subunits, but not in those directed against the S1 subunit (18). Further genomic and antigenicity analysis showed high conservancy (90-100%) of immunogenic epitopes in all the four structural proteins across different SARS-CoV-2 strains, both in T-cell- and B-cell-mediated immunity. These observations provide much hope for a high coverage range of an eventual vaccine, which should be designed to target the conserved epitopes (16). However, this cross-immunity is likely to be insufficient to confer effective therapeutic or preventive effect against the infection (19). Furthermore, additional observations that correlated clinical data with T-cell-mediated immune response against S, M, and N proteins of the virus, which mostly activates CD4+ and CD8+ T cells, suggested that this immune response is associated with hyper-activity and immunopathogenesis, rather than virus neutralization (20).

Thus, the important pending issue to date is to identify the virus epitopes that elicit the production of neutralizing antibodies and to isolate the neutralizing antibodies, which would be of a major therapeutic benefit. As a consequence, there is a growing interest in detecting and monitoring the levels of specific anti-SARS-CoV-2 immunoglobulins in different phases of the disease, and conducting clinical studies to correlate the immunoassay findings with disease severity, outcomes, and reinfections.

In this retrospective cohort, the researchers investigated specific anti-SARS-CoV-2 IgG and IgM antibodies production in 70 serum specimens from 23 rt-PCR-positive severe COVID-19 patients, using both qualitative and quantitative immunoassays. The findings show that IgG production starts early (0-4 days) after the first positive rt-PCR in approximately two-third cases. Both serum concentration and seroconversion rate increased gradually until the latest period, i.e. beyond 15 days, where seroconversion rate reaches 100% of the sera collected. This IgG production was simultaneously detected using the qualitative immunoassays and both quantitative immunoassays, and substantial-to-perfect agreement was found between them. However, IgM detection was inconsistent, roughly following an inverse bell-shaped curve, with slight-to-fair agreement between the two quantitative immunoassays. Furthermore, the researchers observed no significant association between specific IgG and IgM immunoglobulin production and patient mortality in this cohort of severe COVID-19 cases.

Using the qualitative method, namely Abbott SARS-CoV-2 IgG immunoassay, this study found a substantial and gradual increase in seroconversion rates, reaching 90.0% in sera collected 10-15 days after the first positive rt-PCR and 100% in sera collected later. Previous investigations of the qualitative Abbott SARS-CoV-2 IgG test showed sensitivity and specificity as high as 88.0% and 99.6%, respectively, one week after the onset of symptoms, using a cutoff index value of 0.7. The sensitivity of the test gradually increased to reach 94.4% after 10 days and 97.9% after two weeks following symptoms onset, using the same index value cut-off (21). Congruously, both quantitative immunoassays investigated in this study showed that IgG concentration increased gradually over time, reaching maximal levels at the latest period (>15 days after the first positive rt-PCR) with 100% seroconversion rate using the respective positivity cut-off for each assay.

This study showed inconsistent IgM detection, with levels of IgM grossly peaking between 10-15 days after the first positive rt-PCR then decreasing in the latest period. The same observations were reported by some previous studies that showed inconsistent IgM detection in COVID-19 patients, even in the early phases of the disease, while IgG was consistently observed in same patients, notably in the convalescence phase (22). This may be due to IgM production starting earlier before symptoms onset, while serological testing is only carried out at the patient’s presentation or after positive rt-PCR, resulting in a number of patients having already achieved negative IgM seroconversion. Thus, the present study suggests that the kinetics of IgM and IgG production in patients with severe COVID-19 is comparable to that in non-severe cases; this is further demonstrated by the absence of significant association with mortality and the consistency of these observations throughout the immunoassays. However, further investigation is warranted to applicability of such conclusions or the existence of a different clinical-immunological correlation in patients infected with the newly emerging variants of the virus, which were not identified at the time of the present study.

Of note, pairwise comparisons of the two quantitative assays showed higher conversion rates using the ANSH kit compared to the AESKU kit, both in IgG and IgM antibody detection, and which was consistent through time. This difference may be explained by the ANSH kit being more sensitive owing to its two capture antigens, the S and NP antigens, being coated in the ELISA assay, while the AESKU kit is coated only with the NP antigen. This highlights the need for proper selection of the test to be used with regards to the desired level of sensitivity and with respect to the screening strategy.

Whether this specific immunoglobulin production is capable of neutralizing SARS-COV-2 virus or reducing its virulence or infectiveness has not been demonstrated. An *in vitro* study that tested SARS-CoV-2 S1-specific monoclonal IgG antibodies from the sera of 26 convalescent patients showed minor blockade effect on the receptor binding domain of the virus to the host receptor, namely angiotensin conversion enzyme receptor 2 (23). Another study showed that the production of neutralizing antibodies among 175 recovered patients from mild COVID-19 is detected 10-15 days after symptom onset and maintained thereafter; however, the titer of neutralizing antibodies varied significantly between patients and was correlated positively with age and C-reactive protein levels, while it was inversely correlated with lymphocyte count (24).

These observations indicate the reliability of specific IgG serological testing in detecting past exposure to the virus or convalescents that went undiagnosed, in a systematic screening perspective, while being of limited interest in the early diagnosis of the disease.

In the present cohort of hospitalized patients with severe COVID-19, and in the absence of comparative data, the researchers noted high IgG concentrations with substantial increase in seroconversion rates over time, while IgM production was inconsistent and declined over time. This is broadly in line with other case-control studies that demonstrate higher, earlier, and increased anti-S IgG production in hospitalized patients with severe COVID-19 compared to those recovering from mild disease that had delayed seroconversion and relatively lower IgG concentrations (25). However, the same study showed that IgM production declined over time among severe cases, while it gradually increased over the first month following the onset of symptoms (25), which is also commensurate with our findings, which showed decline in IgM in the last period. On the other hand, no significant correlation was found between IgG or IgM concentration and survival outcome, in the presented marked by more than 60% mortality. Conversely, several studies reported an association between high levels of anti-S antibodies and poor clinical outcomes, and correlated delayed and low-to-medium production of neutralizing antibodies with mild and moderate severity of disease (24, 26). Later longitudinal data demonstrated that milder forms of the disease are associated with lower immunoglobulin titers compared to more severe forms. A study by Röltgen et al. analyzed the kinetics of specific IgM, IgG and IgA in the sera of hospitalized versus outpatient COVID-19 patients. By focusing on neutralizing antibodies, i.e. those preventing the interaction of the virus S receptor binding domain (RBD) with the host angiotensin-converting enzyme 2 (ACE2) receptor, authors observed higher antibodies titers among hospitalized patients, with highest titers observed in patients who were admitted in ICU and deceased ones. On the other hand, asymptomatic patients had rapid decline in both IgM and IgG titers. However, authors failed to demonstrate the prognostic value of the early antibody response among hospitalized patients, notably absence of difference in S1 or RBD-specific antibody titers between diseased and survived patients (27). Other interesting data by Gaebler et al. further demonstrated the gradual decrease over time of neutralizing IgM and IgG antibodies with further decrease in neutralizing activity as tested using pseudotype virus assays. This was contrasting with sustained levels of specific memory B cells that were observed in the patients’ sera. Further investigations showed viral antigen persistence in the intestine of infected patients for approximately 6 months, which may explain the sustained levels of specific memory B cells for a comparable period (28). Such observations further indicate the low interest of IgG immunoassays in the early diagnosis of COVID-19, especially for mild cases, as well as in predicting the severity of the disease.

This study may be limited by a small sample size and the variable time of serum specimen collection across patients, which is a product of its retrospective design. These limitations have a limited impact on the interpretation of the findings: most notably affecting the analysis of pooled data, which is based on the assumption of equivalent immune response across individuals. On the other hand, the study used 3 different immunoassays having different capture protein antigens, NP for Abbott and AESKULISA and NP and S for ANSH, and showed a strong agreement between the respective results, notably between Abbott-AESKU IgG levels (R=0.87) and ANSH-AESKU serological statuses (k=0.86). This supports the reliability of the assays and the findings, and constitutes the strength of this study.

In conclusion, patients with severe COVID-19 develop an early, robust anti-SARS-CoV-2-specific humoral immune response, principally involving IgG immunoglobulins production, which reaches substantial plasma concentrations and 100% seroconversion rate two weeks after diagnosis. These findings were concordantly observed using three different commercial immunoassays, including one quantitative method and two qualitative and qualitative methods, showing very high-to-perfect agreement, notably in IgG detection. However, these patients have inconsistent IgM production, which declined 10-15 days after disease onset. The present findings, combined with currently available knowledge of the disease, indicate the low interest of IgM and IgG immunoassays in the early diagnosis of COVID-19 and question their reliability in predicting the severity of the disease or the patient outcome. Furthermore, given the high sensitivity and specificity, IgG immunoassays can be reliably used in detecting past exposure to the virus, notably to screen for undiagnosed convalescents, in an integrative systematic screening strategy combined with rt-PCR. However, the effectiveness and cost-effectiveness of such approach are to be determined according to the objectives of the screening strategy.

## Data Availability Statement

The original contributions presented in the study are included in the article/**Supplementary Material**. Further inquiries can be directed to the corresponding author.

## Ethics Statement

The study protocol was approved by the Research Ethics Committee of King Abdulaziz University (Reference No. 478-20). The patients/participants provided their written informed consent to participate in this study.

## Author Contributions

Guarantor of the article: JA-M. Development of study concept and design: JA-M and OS. Acquisition, analysis, and interpretation of the data: JA-M, TA-M, and OS. Statistical analysis: JA-M and OS. Drafting of the manuscript: JA-M and OS. Critical revision of the manuscript for important intellectual content: JA-M, TA-M, and OS. All authors contributed to the article and approved the submitted version.

## Conflict of Interest

The authors declare that the research was conducted in the absence of any commercial or financial relationships that could be construed as a potential conflict of interest.

## Publisher’s Note

All claims expressed in this article are solely those of the authors and do not necessarily represent those of their affiliated organizations, or those of the publisher, the editors and the reviewers. Any product that may be evaluated in this article, or claim that may be made by its manufacturer, is not guaranteed or endorsed by the publisher.
