# Supplementary material for: Monitoring Specific IgM and IgG Production Among Severe COVID-19 Patients Using Qualitative and Quantitative Immunodiagnostic Assays: A Retrospective Cohort Study
Source: Front Immunol. 2021 Sep 3;12:705441. doi: 10.3389/fimmu.2021.705441 (PMC8446649; doi:10.3389/fimmu.2021.705441)
Supplement: Supplementary file 1 [file DataSheet_1.docx]

**Supplementary materials**

**Supplemental Table 1**: Results of the first quantitative IgM and IgG testing for hospitalized COVID-19 patients.

| **Patient** | **Time from diagnosis (days)** | **IgM** | | | | **IgG** | | | |
| --- | --- | --- | --- | --- | --- | --- | --- | --- | --- |
|  |  | **Method 1 (AESKU)** | | **Method 2 (ANSH)** | | **Method 1 (AESKU)** | | **Method 2 (ANSH)** | |
|  |  | **Titer** | **CCL** | **Titer** | **CCL** | **Titer** | **CCL** | **Titer** | **CCL** |
| 1 | 0 | 1 | - | 2.61 | - | 1.24 | - | 5.74 | - |
| 2 | 1 | 16.1 | + | 5.77 | - | >100 | + | 12.36 | + |
| 3 | 1 | 6.53 | - | 13.38 | + | 53.4 | + | 20.31 | + |
| 4 | 1 | 1.11 | - | 2.95 | - | 10.47 | Eq. | 19.02 | + |
| 5 | 1 | 3.55 | - | 12.12 | + | >100 | + | 41.46 | + |
| 6 | 2 | 1.35 | - | 6.17 | - | 5.35 | - | 3.75 | - |
| 7 | 2 | 1 | - | 2.95 | - | 1 | - | 1.36 | - |
| 8 | 2 | 5.53 | - | 19.1 | + | >100 | + | 222.49 | + |
| 9 | 3 | 1.18 | - | 2.49 | - | 1.08 | - | 6.11 | - |
| 10 | 3 | 1.93 | - | 8.87 | - | >100 | + | 244.39 | + |
| 11 | 3 | 1 | - | 6.54 | - | 1.57 | - | 10.97 | Eq. |
| 12 | 4 | 4.5 | - | 18.49 | + | >100 | + | 2754.3 | + |
| 13 | 5 | 1.13 | - | 3.6 | - | 1.42 | - | 6.53 | - |
| 14 | 5 | 3.55 | - | 2.62 | - | 1.15 | - | 3.19 | - |
| 15 | 10 | 1 | - | 4.78 | - | 1.58 | - | 3.52 | - |
| 16 | 10 | 3.07 | - | 11.45 | Eq. | 10.51 | Eq. | 111.8 | + |
| 17 | 14 | 1.71 | - | 8.37 | - | >100 | + | 225.31 | + |
| **Positive conversion rate** | |  | **5.9%** |  | **23.5%** |  | **41.2%** |  | **52.9%** |

Result: + positive, - negative, Eq. equivocal.

**Supplemental Table 2**. Comparison of IgM and IgG titers and levels between diseased and recovered patients

| **Mean Time from PCR** | **Antibody, method** | **Recovered** | | **Deceased** | | **p-value** |
| --- | --- | --- | --- | --- | --- | --- |
|  |  | **Median** | **P75** | **Median** | **P75** |  |
| 4 days | IgG, ABBOTT | 0.47 | 2.95 | 0.13 | 3.77 | .688 |
|  | IgG, AESKU | 10.49 | 65.05 | 1.57 | 100.00 | .779 |
|  | IgG, ANSH | 19.67 | 144.94 | 6.53 | 12.36 | .336 |
|  | IgM, AESKU | 1.56 | 3.93 | 1.13 | 5.53 | 1.000 |
|  | IgM, ANSH | 5.91 | 11.93 | 4.78 | 6.54 | .779 |
| 10 days | IgG, ABBOTT | 4.91 | 7.09 | 5.26 | 7.42 | .688 |
|  | IgG, AESKU | 100.00 | 100.00 | 100.00 | 100.00 | .918 |
|  | IgG, ANSH | 143.20 | 859.54 | 213.70 | 2754.30 | .758 |
|  | IgM, AESKU | 8.92 | 31.48 | 9.44 | 26.43 | .758 |
|  | IgM, ANSH | 22.74 | 27.55 | 12.71 | 18.04 | .536 |
| 16 days | IgG, ABBOTT | 7.15 | 8.07 | 6.61 | 7.20 | .657 |
|  | IgG, AESKU | 100.00 | 100.00 | 100.00 | 100.00 | .864 |
|  | IgG, ANSH | 169.77 | 876.16 | 219.61 | 2754.30 | .776 |
|  | IgM, AESKU | 13.15 | 34.99 | 4.13 | 40.63 | .864 |
|  | IgM, ANSH | 12.05 | 32.36 | 12.41 | 19.37 | .689 |

Test used: Mann-Whitney test.

**Supplemental Figure 1**. Change over time of IgG titers in recovered versus deceased patients using pooled data


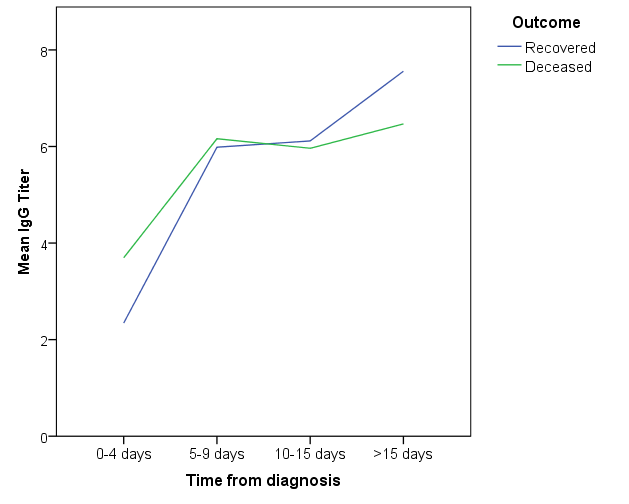


**Supplemental Figure 2**. Change in seroconversion rate by respect of ABBOTT IgG qualitative testing in recovered versus deceased patients


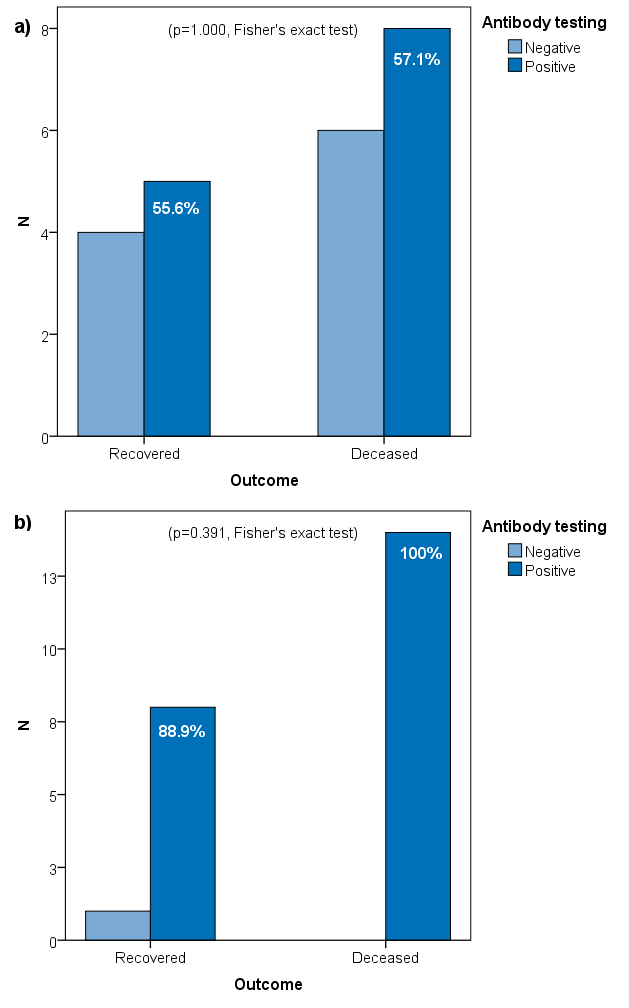


Darker bars represent the proportion of positive seroconversion among recovered versus deceased patients at the first (upper panel a) and second (lower panel b) testing.
